# Notes on Hyperidrosis

**Published:** 1885-09

**Authors:** Henry Wile

**Affiliations:** Atlanta, Ga., Late Clinical Assistant in Dermatology in the University of Pennsylvania


### NOTES ON HYPERIDROSIS.*

BY HENRY WILE, M. D., ATLANTA, GA., LATE CLINICAL ASSISTANT IN DERMATOLOGY IN THE UNIVERSITY OF PENNSYLVANIA.

It is a peculiar fact that diseases of the skin, as regards their therapeutics, have been viewed differently from diseases of other organs of the human body. This was one of the outgrowths of those old and obsolescent theories in pathology, according to which, diseases of the skin were the means whereby nature attempted to throw off certain exudates or morbid secretions coming from the blood. Thus a cessation of the process, almost regarded as physiological, was attended with danger, namely, of driving the poisonous material back into the system, where it might work mischief in one or other organ. Diseases of the skin were, therefore, looked upon in a *noli me tangere* light, and their cure was, for a long time, regarded with suspicion. However, under the light of modern pathological investigation these ideas are well-nigh abandoned. Diseases of the skin have been closely studied, classified and compared with morbid changes occurring in other organs; the chemistry of the secretions and excretions of the body is well understood, and the therapeutics of cutaneous affections has, in consequence, been remarkably developed.

It is my purpose, in this paper, to present briefly a few points of practical interest in connection with a disease of the skin which is often met with in private practice, and is in many cases a source of great annoyance. The disease is called Hyperidrosis (excessive sweating), and belongs to the class of cutaneous affec-

*Presented to the Medical Association of Georgia at its Thirty-Sixth Annual Session, by James A. Gray, M. D.

tions known as anomalies of secretion. The secretion of the sweat glands comes from arterial blood which circulates in a complicated plexus around each glandular coil. It has an acid reaction when fresh, contains about 99 per cent. water, 1 per cent. solid constituents, of which the chief ingredient is chloride of sodium. The quantity of the secretion depends upon the action of the heart, whereby the cutaneous blood-vessels become more or less congested. This may be effected by violent exercise, dancing, etc. The nerves, especially the vaso motor nerves, also have more or less influence. These are usually excited through emotions of various kinds, fright, anxiety, even intense pain. Under these causes the secretion may become excessive, but at the same time be nothing morbid and, therefore, not strictly coming under the term hyperidrosis. On the other hand the condition of excessive sweating common to acute fevers, tuberculosis, etc., are also not included. The term hyperidrosis is used to express that condition in which there is *primarily* an unusual and more or less chronic functional activity of the sweat glands, whereby the quantity of the secretion is increased.

There are two forms called respectively *hyperidrosis universalis* and *hyperidrosis localis*.

Hyperidrosis universalis is met with in middle age or later years of life, also in infancy. When the sweating is profuse it gives rise to sudamina, especially in the delicate skin of the infant.

The treatment of this form is simple, and consists in the use of cooling lotions, such as equal parts of alcohol and rosewater, followed by a powder of starch, or equal parts of starch and oxide of zinc.

Hyperidrosis localis usually affects the face, scalp, genitals, axillæ, palms and soles—one or several of these localities, the last three being the favorite seats. It is mostly encountered in the young of both sexes, and is generally associated with an anæmic, chlorotic, or debilitated condition, though sometimes, but exceptionally, in apparently healthy individuals. The disease is often a source of discomfort, even distress, and when affecting the palms of the hands it incapacitates the individual for various kinds of special work. The palms have a cool, moist, clammy feeling, necessitating frequent drying and, under social circumstances, occasioning deep embarrassment. In the axilla, where the surface is covered, and there is not so much chance for evaporation, the perspiration is confined, staining the garments in the neighborhood, and, undergoing decomposition, acquires a peculiar odor.

The same is true in regard to the plantar surfaces of the feet. The secretion is confined by the socks and shoes, causing more or less maceration of the epidermis, which leads to an exfoliation of the epidermal layers and formation of fissures. This renders the parts tender and painful.

Where profuse sweating continues for a time, and is neglected, there soon developes the condition known as *bromidrosis*. The secretion more or less absorbed by the horny layers of the epidermis, and soaking into the leather of the shoes, undergoes chemical decomposition and emits a disagreeable odor. For it has been shown (Neumann, Lehrbuch der Hautkrankheiten, 1880) that if the soles of the feet are kept scrupulously clean, the secretion of the sweat glands will have no odor. Where, therefore, the disease is allowed to develop, or continue without interference, the secretion quickly undergoes decomposition and the patient is obliged to wash the feet and change socks several times daily, or go around enveloped in an odoriferous cloud. Thus it becomes a loathsome affection.

There are several other forms of local hyperidrosis, in which special regions are affected, as one side of the body (unilateral), or along special nerve tracks. These forms are, however, obscure.

The prognosis in all cases of hyperidrosis is favorable. Most cases are amenable to treatment, and a cure or great relief can always be promised.

In the matter of treatment, one of the first injunctions to the patient is cleanliness. The milder forms, affecting the axillary, genital regions, palmar and plantar surfaces, usually yield to simple measures, such as the use of astringent washes and powders.

Tannic acid ten to thirty grains to a half pint of water; extract aconite ten grains to alcohol and ether each four ounces; a saturated solution of boracic acid; naphtol ten to forty grains to the ounce, of deodorized alcohol, will all be found of value. Painting the parts with pure tincture of belladonna often acts happily. Bulkley recommends for most cases chloral, one ounce to the pint of water. These lotions should be applied freely for three to ten minutes at a time, two or three times a day, according to the severity of the case, each application being followed by the use of some absorbent powder. The following may also be found to be of advantage :

Salicylic acid ten grains to the ounce of starch; burnt alum onc drachm to salicylic acid and venetian talc, each two ounces. Where the skin is in apposition, as in the groins or between the toes, the powder should be used with absorbent cotton. Sometimes a case will resist ordinary measures, and, being obstinate, will require persistent systematic treatment.

In hyperidrosis of the soles, when the simple means indicated above fail to effect a cure, the diachylon ointment treatment, as originally recommended by Hebra, will give satisfaction. Diachylon ointment is composed of litharge and olive oil, in the proportion of one part to four. They are mixed and allowed to boil slowly to the consistency of an ointment, after which oil of lavender is added, in quantity one-tenth the amount of litharge. The ointment is spread on linen and applied to the soles of the feet, which should be previously washed and carefully dried. The plasters may be kept in position by means of a thin gauze bandage or socks. The application ought to be made twice daily for fourteen days, each time the surface of the skin being rubbed gently with dry, absorbent cotton. It is better for the patient to keep a recumbent position during the treatment, or walk as little as possible. Water is also to be kept from the parts during the treatment. The old and diseased epidermis gradually exfoliates, and a new, healthy surface is exposed. The use of some absorbent powder should be kept up a few days longer. Occasionally the procedure may have to be repeated, but by this means a thorough cure can be effected.

The internal treatment of hyperidrosis consists in the administration of tonics, where these are indicated for the correction of any debilitated condition of the system. For its direct imhibitory action upon the secretory apparatus of the skin, atropia in gradually increasing doses has been recommended.

